# Seven Glimpses into the Emotional Brain

**DOI:** 10.1371/journal.pbio.2001633

**Published:** 2016-12-19

**Authors:** Gabriel Gasque

**Affiliations:** Public Library of Science, San Francisco, California, United States of America

In the last century, and particularly since the 1950’s, we’ve witnessed a shift in our efforts to understand the human mind. Cognition—the term that includes mental processes such as attention, memory, problem-solving, planning, and language—stopped being exclusively a philosophical subject approached by introspection and became a matter of empirical investigation and objective quantification. However, the traditional approaches to study cognition have focused on the information-processing functions and have generally excluded emotions.

Today researchers acknowledge a close relationship between our feelings and our thoughts, and have begun to disentangle how this relationship affects how we view the world, what we learn from it, and how we adjust our behavior accordingly. In this short article, I summarize recent research published in Open Access journals that explores the quantification and manipulation of emotions and investigates the interactions between emotions and cognitive processes such as attention and memory. This work provides insights from human subjects and animal models on the underlying neural mechanisms of cognition.

In a recent study published in *PLOS Biology* [1], the authors investigated how emotions impact visual attention. To do so, they used a combination of psychophysical experiments and functional Magnetic Resonance Imaging (fMRI), which measures brain activity by detecting changes associated with blood flow. When human subjects were shown emotionally loaded stimuli, the size of the attention field changed depending on whether the stimuli had a positive or negative valence. For example, a smiling face has a positive valence, and when presented to the subject, their attention field broadened. However, when the subjects were presented with a face with a negative valence (frowning or angry), the subject’s attention field narrowed. Interestingly, the emotional strength of the negative or positive faces, as explicitly reported by the participants, closely matched the size of the effects in the behavioral and brain-imaging experiments. The authors could establish that the effects of emotional valence on the size of the attention fields were mediated by a feedback from the dorsolateral prefrontal cortex—a functional brain structure that contributes to executive functions such as working memory, cognitive flexibility, planning, and abstract reasoning—to the primary visual cortex. These findings pave the road for understanding how the interaction between emotion and attention shapes our experience of the world.

In a complementary study published in *PLOS Computational Biology* [2], researchers explored additional neural mechanisms that could account for the emotional regulation of attention. The scientists developed a computational model to investigate how the amygdala—the gateway of the brain's emotional system—controls the inhibitory thalamic reticular nucleus, which is central to the attentional system. Lesions in the thalamic reticular nucleus, for example, result in attentional deficits. The computational model indicates that the amygdala-thalamic reticular nucleus pathway can select emotionally salient stimuli and inhibit transmission of irrelevant signals to the cortex, thereby determining the information available for subsequent cortical processing. In this way, the model accounts for both flexible bottom-up and focused top-down attention guided by emotions. Bottom-up (or stimulus-driven) attention concentrates on the most important aspects of the environment to drive adaptive behaviors. Top-down (or goal-directed) attention focuses only on what is relevant according to a pre-selected plan. Importantly, the model predicts three mechanisms that could potentially contribute to abnormal emotional-related behavior: dysfunctional cortical drive to the amygdala and dysregulation of local inhibition in the amygdala as well as in the cortex.

Emotions influence not only the way we perceive, view, and react to environmental stimuli, but also how we learn and create new memories. A recent study published in *Nature Communications* [3] shed light onto the neurobiological mechanisms that underlie the effects of emotions on learning. Experiments carried out in mice revealed that a single synaptic connection between the amygdala and the ventral hippocampus—a region of the brain involved in both processing emotions like anxiety and fear as well as in regulating learning and memory—could explain the influence of emotions on learning. The authors showed that opposite emotional states—helplessness and hopefulness—modulate in different directions both spatial memory formation and the strength of amygdala-to-hippocampus synaptic connectivity. Hopefulness enhanced spatial memory and increased the synaptic connection via the up-regulation of excitatory receptors known as AMPA receptors. Conversely, helplessness induced memory deficits and attenuated the synaptic connections. To prove that this synaptic connection was causative, the authors artificially weakened the connection between the amygdala and the hippocampus in hopeful mice or strengthened the connection in helpless mice, and found they were able to eliminate the expected effect. Disruption of the synaptic connection impaired hopefulness-induced synaptic plasticity and abolished the positive effects on memory; whereas experimentally stimulating the amygdala-to-hippocampus synapse rescued helplessness-induced memory deficits and mimicked the effects of hopefulness.

**Fig 1:**
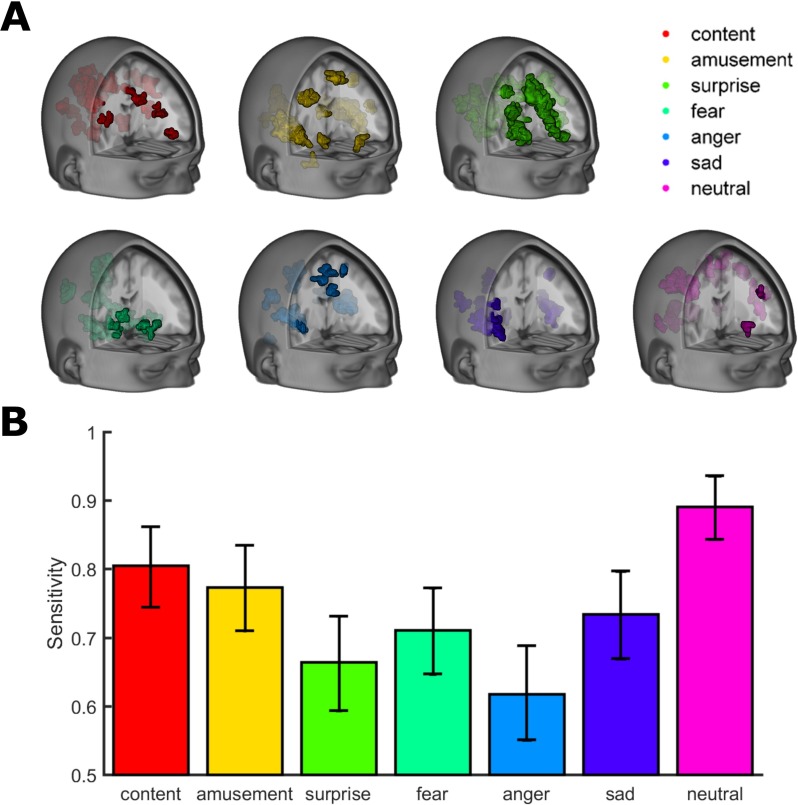
(A) Parametric maps indicate brain regions in which increased fMRI signal informs the classification of emotional states. (B) Sensitivity of the seven models. Error bars depict 95% confidence intervals. *Image credit*: *doi*:10.1371/journal.pbio.2000106

To better understand emotions and their influences on cognition, researchers need a way to measure emotions objectively, without relying on a subject’s description of their emotional state. In a recent study published in *PLOS Biology* [4], the authors used fMRI to correlate different patterns of activation of the whole brain with different categories of emotions (Fig 1). By treating the fMRI signal like a problem of pattern recognition—similar to face- or character-recognition software—the authors could classify different patterns of activation as different emotional states, like *content*, *amusement*, *surprise*, *fear*, *anger*, *sadness*, or *neutral*. The authors could then tell the emotional state of the participants and could successfully predict their feelings in a resting mind-wandering state. Interestingly, the data showed that an emotional state was not stable, instead the brain visited multiple distinct emotional states during the interval that the experiment lasted. These fluctuations among distinct feelings depended on the emotional status of the subject: individuals with traits of depression or anxiety, visited *sadness* and *fear*, respectively, more frequently. The authors argue that their findings are relevant to clinical settings, suggesting that the emotional brain-decoding tool may be useful to assess the emotional status of patients who cannot report their own feelings, like patients in a locked-in state, for example.

Think of patients suffering from amyotrophic lateral sclerosis (ALS), sometimes called Lou Gehrig's disease. ALS is a rapidly progressive, invariably fatal multi-system disorder that is mainly characterized by degeneration of the motor-neurons in the brain and spinal cord that control voluntary movements. While classically seen as a motor disease that eventually traps patients in a completely locked-in state, ALS also impairs cognitive and emotional processes. An article in *PLOS ONE* [5] documents that these patients showed decreased behavioral responses in processing of disgust and fear, because they were less accurate at recognizing faces expressing those emotions. In a complementary brain imaging experiment using fMRI, the patients suffering from ALS showed altered activity in areas involved in processing negative emotions such as sadness. The authors argue that this decrease in negative emotions might be adaptive to the negative consequences of neurodegenerative processes associated with the fatal disease.

Adaptive emotional responses require healthy underlying neuronal tissue. Research published in *Nature Communications* [6] explored the mechanisms required for normal development of the amygdala. Ephrins are membrane-bound proteins that act as ligands of Eph receptors and have been extensively documented as fundamental for axon guidance. This study found that ephrin-B3 is required during a critical period in the neonatal brain for axon targeting and dendritic spine formation in the amygdala and for the development of innate fear. The authors argue that because aberrant wiring of emotional circuits could lead to neurodevelopmental disorders characterized by socio-emotional impairments, these results could explain why deficiencies in Ephrin-B/EphB signaling have been linked to anxiety disorders and autism.

A fundamental challenge for future studies, particularly in humans, is to develop strategies to understand the mechanistic underpinnings of emotions and their influence on cognition. This would require the experimental manipulation of the distributed networks that support these mental processes. In a study published in *PLOS Biology* [7], scientists demonstrate that inducing specific patterns of activity within the same specific brain region can lead to opposite emotional states. The authors used a technique called *fMRI decoded neurofeedback* to manipulate the activity in the cingulate cortex, a part of the cerebral cortex that is important for establishing preferences to different categories. As the participants looked at faces that were neutrally preferred, *fMRI decoded neurofeedback* was used to induced patterns of brain activity that corresponded to higher (or lower) preference. This perturbation resulted in the neutrally preferred faces becoming more (or less) preferred, without the subject being aware of the aim to change the preference. These exciting results reveal that highly specific patterns of activation within a brain region, rather than total activation of that region, are important for cognitive functions; in this case, positive and negative facial preferences are represented by spatially overlapping but distinct activation patterns in the cingulate cortex. The experimental activation of these specific patterns suffices to induce a change in preferences beyond the subject’s will.

For more detailed reading please see the associated PLOS Collection [8].
